# Predictors of Hypocalcemia after Thyroidectomy: Results from the Nationwide Inpatient Sample

**DOI:** 10.5402/2012/838614

**Published:** 2012-07-15

**Authors:** Randall L. Baldassarre, David C. Chang, Kevin T. Brumund, Michael Bouvet

**Affiliations:** Department of Surgery, School of Medicine, University of California San Diego, San Diego, CA 92093-0987, USA

## Abstract

Hypocalcemia is a common complication following thyroidectomy. However, the incidence of postoperative hypocalcemia varies widely in the literature, and factors associated with hypocalcemia after thyroid surgery are not well established. We aimed to identify incidence trends and independent risk factors of postoperative hypocalcemia using the nationwide inpatient sample (NIS) database from 1998 to 2008. Overall, 6,605 (5.5%) of 119,567 patients who underwent thyroidectomy developed hypocalcemia. Total thyroidectomy resulted in a significantly higher increased incidence (9.0%) of hypocalcemia when compared with unilateral thyroid lobectomy (1.9%; *P* < .001). Thyroidectomy with bilateral neck dissection, the strongest independent risk factor of postoperative hypocalcemia (odds ratio, 9.42; *P* < .001), resulted in an incidence of 23.4%. Patients aged 45 years to 84 years were less likely to have postoperative hypocalcemia compared with their younger and older counterparts (*P* < .001). Hispanic (*P* = .003) and Asian (*P* = .027) patients were more likely, and black patients were less likely (*P* = .003) than white patients to develop hypocalcemia. Additional factors independently associated with postoperative hypocalcemia included female gender, nonteaching hospitals, and malignant neoplasms of thyroid gland. Hypocalcemia following thyroidectomy resulted in 1.47 days of extended hospital stay (3.33 versus 1.85 days *P* < .001).

## 1. Introduction

Postoperative hypocalcemia is a common complication following thyroidectomy. Decreased serum calcium, secondary to hypoparathyroidism, may present clinically with muscle cramps, perioral and peripheral paresthesias, carpopedal spasm or tetany, and/or confusion. Symptomatic patients often require extended hospitalizations following thyroid surgery, leading to increased healthcare costs [1]. Depending on the extent of parathyroid gland damage, hypocalcemia may be transient, resolving within a few months, or permanent, requiring lifelong oral calcium and vitamin D supplementation. 

Particularly with ambulatory thyroid surgery, which allows for early discharge of patients, postoperative hypocalcemia is an important consideration. In fact, some surgeons advocate indiscriminate postoperative calcium supplementation, though this approach has been contested [2]. The interest in outpatient and short-stay thyroid surgery makes it especially helpful for surgeons to be able to identify patients at risk of developing hypocalcemia [3, 4]. Therefore, accurate and standardized outcomes data following thyroidectomy are essential [5, 6].

Unfortunately, although hypocalcemia is well documented in the thyroid surgery literature, there are significant limitations to the results of previous studies. Perhaps most noteworthy, the reported hypocalcemia incidence rates range widely; studies report that anywhere from 0.3%–66.2% of patients develop hypocalcemia after thyroid surgery [1, 7]. Part of this variation in incidence is likely related to the fact that reports differ in the thyroid surgery procedure assessed. Some studies, for example, include patients who underwent not only total thyroidectomy, but also less extensive procedures with relatively low risk of hypocalcemia, such as thyroid lobectomy [8]. Such reporting can underestimate the incidence of hypocalcemia and may lead to misinterpretation.

Much of the variability among results may also be attributed to the numerous clinical definitions of hypocalcemia used at different institutions. For instance, some authors consider hypocalcemia to be the clinical presentation of symptoms, whereas others document hypocalcemia on the basis of serum thresholds alone [9, 10]. To demonstrate the effect of relying on the literature to compare outcomes, the authors of one recent study showed that their thyroidectomy patient cohort had a postoperative hypocalcemia rate ranging from 0–46%, depending on 10 different definitions of hypocalcemia adopted by previous studies [11]. The discrepancies that result from these definitions clearly hinder the clinical relevance of thyroidectomy outcome studies.

Without reliable parameters, the relative risk of thyroidectomy cannot be properly assessed for informed consent, and outcomes research lacks a standard benchmark for comparison. Numerous small studies have attempted to identify predictors of postoperative hypocalcemia (12–15), but if the results of such studies are to influence the surgical management of patients at other institutions, a standard definition of hypocalcemia is essential. Alternatively, a population-based study of hypocalcemia can address the limitations of series reports.

The current literature indicates that the incidence of hypocalcemia may be affected by a number of risk factors. More extensive thyroidectomy procedures, for instance, result in a greater incidence of hypocalcemia, though the exact mechanism behind the association is unclear [12]. Other potentially predictive variables of postoperative hypocalcemia, such as iatrogenic parathyroidectomy, were not demonstrated to have an effect on patient outcomes [13]. Some experts posit that a surgeon's skill and experience affect the occurrence of postthyroidectomy hypocalcemia [3, 14]. However, one study found that patients operated on by surgical trainees had complications that were comparable to patients operated on by consultant surgeons [15].

Given that most studies exploring parameters associated with complications after thyroid surgery have small sample sizes and include limited procedures, risk factors are still largely debatable. Few studies have conducted population-based analyses of thyroidectomy outcomes [16–19]. To date, no such study has identified national risk factors of thyroid surgery complications.

To better study the problem of postoperative hypocalcemia following thyroidectomy, we utilized the Nationwide Inpatient Sample (NIS), Healthcare Cost and Utilization Project (HCUP), Agency for Healthcare Research and Quality. The NIS is the largest all-payer inpatient care database in the United States, containing data on more than seven million hospital stays from approximately 1,000 hospitals. Its large sample size is ideal for developing national and regional estimates and enables analyses of rare conditions, uncommon treatments, and special populations [20]. The trends revealed by NIS may be incorporated into informed consent forms and preoperative discussions, allowing both patients and surgeons to make decisions based on the collective experience throughout the United States rather than institution-specific result.

## 2. Materials and Methods

The NIS database was queried for all patients undergoing thyroid surgery from 1998–2008, including the following surgical procedures: total thyroidectomy, unilateral thyroid lobectomy (includes hemithyroidectomy), complete substernal thyroidectomy, substernal thyroidectomy, partial substernal thyroidectomy, isthmectomy, partial thyroidectomy, and thyroidectomy plus bilateral or unilateral neck dissection (International Classification of Diseases, Ninth Revision [ICD-9] procedure codes 06.2, 06.39, 06.4, 06.5, 06.51, 06.52, 40.41, 40.42). From this list of patients, the specific outcome examined was postoperative hypocalcemia, defined as assignment of the corresponding diagnostic code (ICD-9 code 275.41). Patients with thyroid neoplasms were identified and distinguished as having either benign (ICD-9 code 226) or malignant (ICD-9 code 193) disease. Additional inpatient variables extracted included age, gender, ethnicity, length of hospital stay, and teaching hospital status.

Statistical analysis was conducted using Stata, version 9.2 (Stat Corp, College Station, Texas), with statistical significance defined as *P* < .05. The primary measured outcome in the NIS dataset was in-hospital, postoperative hypocalcemia. Univariate analysis was performed using chi-square tests for categorical data and unpaired *T*-tests for continuous data. Variables associated with postoperative hypocalcemia by univariate analysis were incorporated into multivariate logistic regression to identify independent risk factors of postoperative hypocalcemia following thyroidectomy. Odds ratios were expressed relative to a reference baseline category.

## 3. Results

A total of 119,567 thyroidectomy patients were identified in the NIS from 1998–2008. Postoperative hypocalcemia occurred in 5.5% (*n* = 6,605) of all thyroidectomy patients before discharge. The inpatient demographic characteristics and clinical characteristics are summarized in Table 1. Of the patients with recorded gender and ethnicity, most were female (81.1%) and white (71.1%). Roughly half (49.3%) the patients were diagnosed with a neoplasm of the thyroid gland, classified as either malignant (61.6%) or benign (38.4%). 

The most common procedures were total thyroidectomy (39.0%) and unilateral thyroid lobectomy (38.5%). Among the 3,309 patients treated with thyroidectomy plus neck dissection, 2,587 (78.2%) underwent unilateral dissection and 722 (21.8%) underwent bilateral dissection. On average, patients undergoing thyroidectomy had a hospital length of stay of 1.9 days. 

Patients undergoing total thyroidectomy had a postoperative hypocalcemia incidence of 9.0%, compared with 1.9% following unilateral thyroid lobectomy, 14.4% following thyroidectomy plus unilateral neck dissection, 23.4% following thyroidectomy plus bilateral neck dissection, 9.6% following complete substernal thyroidectomy, 3.4% following partial substernal thyroidectomy, 6.5% following substernal thyroidectomy, and 3.4% following isthmectomy or otherwise unspecified partial thyroidectomy (*P* < .001, Table 2). The hypocalcemic group had younger patients (*P* < .001), more females (*P* < .001), and more diagnoses thyroid gland malignancies (*P* < .001). Postoperative hypocalcemia was also more likely to occur at nonteaching hospitals (*P* = .008). Mean hospital stay was 3.33 ± 3.09 days for hypocalcemic patients and 1.85 ± 3.55 days for normocalcemic patients (*P* < .001). 

Results of the multiple logistic regression analysis are summarized in Table 3. The type of operative procedure was independently associated with hypocalcemia, with total thyroidectomy (OR = 3.72, 95% CI 3.27–4.24), thyroidectomy plus unilateral neck dissection (OR = 5.58, 95% CI 4.65–6.70) or bilateral neck dissection (OR = 9.42, 95% CI 7.40–11.99), and complete substernal thyroidectomy (OR = 3.54, 95% CI 2.70–4.66) resulting in an increased risk of hypocalcemia when compared with unilateral thyroid lobectomy (*P* < .001). Of all risk factors, thyroidectomy plus bilateral neck dissection was most likely to result in postoperative hypocalcemia. Female gender, nonteaching hospital, and thyroid gland malignancy also emerged as independent predictors of postoperative hypocalcemia, whereas older age had a protective effect. Each one-year increase in age was associated with a 1.0% decreased risk of postoperative hypocalcemia (OR = 0.990, 95% CI 0.988–0.992, *P* < .001).

When compared with white patients, Hispanic (OR = 1.20; 95% CI 1.06–1.36, *P* = .003) and Asian or Pacific Islander (OR = 1.21, 95% CI 1.02–1.44, *P* = .027) patients were more likely, and black patients were less likely (OR = 0.77, 95% CI 0.64–0.91, *P* = .003) to develop hypocalcemia. 

**Figure 1:**
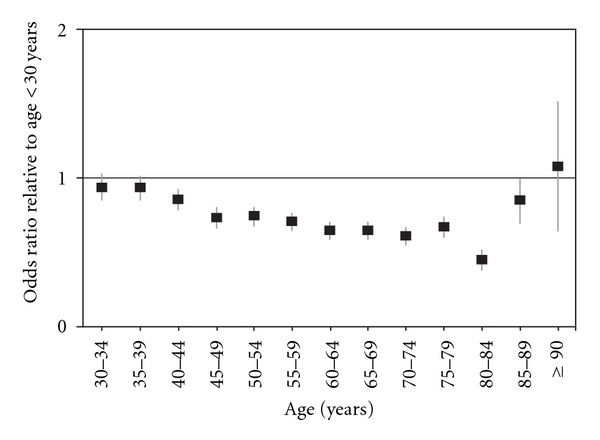
Multiple logistic regression analysis to examine the incidence of postoperative hypocalcemia among thyroidectomy inpatients by age.

Figure 1 illustrates the ORs of postoperative hypocalcemia in 5-year age groups relative to patients younger than 30 years. Although patients between the ages of 30 years and 44 years had similar rates of hypocalcemia when compared with younger patients, individuals aged 45 years to 84 years had significantly lower likelihoods of developing hypocalcemia. Patients 85 years or older had an incidence of postoperative hypocalcemia that was statistically indistinguishable from that of the youngest group.

## 4. Discussion

This study is among the first to report national trends in the incidence of postoperative hypocalcemia following thyroid surgery. We found that the incidence of hypocalcemia after all categories of thyroidectomy was 5.5%. Nearly one-quarter of thyroidectomy patients requiring bilateral neck dissection developed hypocalcemia, the highest incidence of all groups analyzed. Owing to the paucity of larger studies with patients grouped by clinical characteristics or demographics, it is difficult to make direct comparisons of thyroid surgery outcomes. Most published series include a large proportion of total thyroidectomy patients, among whom the incidence of hypocalcemia was 9.0% in the present study. The few studies conducted with large populations, for instance, one international study [18] and another reporting on outcomes throughout Maryland [19], similarly reported that approximately one-tenth of bilateral thyroid surgery cases resulted in hypocalcemia (Table 4). 

As expected, the results of our analysis demonstrate that, on a population level, the incidence of hypocalcemia depends on the extent of surgery. Consistent with the existing body of literature [3, 21], hypocalcemia occurred significantly more often after total thyroidectomy than after unilateral thyroid lobectomy. Patients treated by thyroidectomy with concomitant neck dissection were more likely to develop hypocalcemia than patients who underwent total thyroidectomy alone, supporting the results of smaller series [22]. Incidental parathyroidectomy is believed by many to explain the increased risk of hypocalcemia with more extensive and bilateral surgery [1, 23]. The same trend is seen in past studies for other common, procedure-related complications such as recurrent laryngeal nerve palsy [24]. The 1.9% hypocalcemia incidence associated with less extensive lobectomy procedures was likely attributable to reoperations, though we did not have data on patient surgical history to further analyze this finding.

The higher incidence of hypocalcemia among thyroid cancer patients is consistent with previous studies [19, 25, 26]. Some authors believe that malignancy tends to be treated with a more aggressive approach to thyroid surgery, thereby leading to incidental parathyroidectomy and hypocalcemia [26]. Indeed, neck dissection, which is indicated for thyroid cancer patients with lymph node metastasis, was the strongest risk factor for postoperative hypocalcemia in our analysis. Nonetheless, malignancy emerged as an independent predictor. The altered anatomy of these patients could increase the likelihood of parathyroid tissue removal. The association between postoperative hypocalcemia and female gender also found in the literature, may be due to women being more prone to calcium and vitamin D deficiency than men [18, 27].

Our data reveal a statistically elevated risk of hypocalcemia for patients younger than 45 years compared with their older counterparts. The protective effect against hypocalcemia increases with age, a trend demonstrated in Figure 1, though patients 85 years and older fared the same as the youngest patients. Although the inverse association between hypocalcaemia and advancing age corroborates the work of others [27, 28], there is some conflict found in the literature. A previous study that also assessed the NIS, though for a narrower time frame, from 2003-2004, showed that patients 65 years and older, after adjusting for other risk factors, experienced similar rates of hypocalcemia and recurrent laryngeal nerve injury compared with younger patients [29]. In fact, older patients in the study had higher rates of total complications, consistent with a recent series [30]. It was previously demonstrated that younger patients may be more likely to undergo bilateral surgery for malignancy, which would expectedly result in an association between young age and hypocalcemia [25]. However, our data reveal an increased risk of hypocalcemia among younger patients irrespective of malignancy. 

The effect of ethnicity on hypocalcemia revealed by our analysis has not been demonstrated in the literature. Rather, thyroidectomy outcome studies reveal no difference in rates of hypocalcemia by race. One NIS study found that black patients undergoing thyroid procedures experienced higher overall complication rates following thyroid surgery compared with white or Hispanic patients, but the complications were not endocrine specific [31]. We cannot explain the better outcomes for black patients and worse outcomes for Hispanic, Asian, and Pacific Islander patients compared with white patients in our study, and these new findings deserve further investigation. 

There are some limitations to our study. We were unable to identify which patients went on to develop permanent, as opposed to transient, hypocalcemia. The NIS database also does not make it possible to identify patients that actually developed symptoms related to their low postoperative calcium levels. The longer hospital stay for hypocalcemic patients, however, provides evidence of the clinical consequences of this common postoperative complication. 

Postoperative hypocalcemia should clearly be considered in discussions concerning the appropriate extent of thyroid surgery. Particularly with more extensive resections, surgeons may take corrective measures to reduce the incidence of hypocalcemia and improve long-term outcomes. For instance, if incidental excision or devascularization of the parathyroid glands is noted, parathyroid autotransplantation may reduce the occurrence of permanent hypocalcemia among patients with postoperative hypocalcemia [23, 32, 33]. 

In summary, postoperative hypocalcemia is a multifactorial, problematic source of morbidity among thyroid surgery patients, resulting in longer hospital stays and thereby increased procedure costs. Interventions such as routine oral calcium and/or vitamin D supplements have been shown to reduce the incidence of hypocalcemia [34] and may be particularly beneficial in patients at risk, including those with malignancy and/or individuals undergoing total thyroidectomy. Outpatient total thyroidectomy studies that already adopted this preventive approach showed comparatively low rates (5.1%) of hypocalcemia [35]. 

With the use of extensive and ambulatory thyroid surgery to treat benign conditions such as multinodular goiter, close monitoring of patients at risk for the development of hypocalcemia is particularly warranted. Future studies should further investigate newly identified and controversial predictors of postoperative hypocalcemia, including ethnicity and younger age, with the goal of ultimately reducing the incidence, cost, and long-term effects of this common complication.

## Figures and Tables

**Table 1:** Thyroidectomy patient demographics and clinical characteristics.

Variable^a^	Value
Overall	119,567
Age, mean (SD), *y*	50.7 (15.9)
Gender	118,375
Female	96,012 (81.1)
Male	22,363 (18.9)
Ethnicity	91,134
White	64,808 (71.1)
Black	11,422 (12.5)
Hispanic	7,848 (8.6)
Asian or Pacific Islander	3,855 (4.2)
Native American	354 (0.4)
Other	2,847 (3.1)
Principal procedure	119,567
Total thyroidectomy	46,630 (39.0)
Unilateral thyroid lobectomy	46,046 (38.5)
Thyroidectomy with unilateral neck dissection	2,587 (2.2)
Thyroidectomy with bilateral neck dissection	722 (0.6)
Complete substernal thyroidectomy	2,959 (2.5)
Partial substernal thyroidectomy	1,598 (1.3)
Substernal thyroidectomy, not otherwise specified	108 (0.1)
Other: isthmectomy, partial thyroidectomy not otherwise specified	18,917 (15.8)
Length of stay, mean (SD), *d*	1.9 (3.5)
Hospital type	119,483
Teaching	67,134 (56.2)
Nonteaching	52,349 (43.8)
Neoplasm of thyroid gland	58,931
Malignant	36,278 (61.6)
Benign	22,653 (38.4)

^a^Continuous variables are presented as mean (SD); categorical variables are presented as *n* (%).

**Table 2:** Comparison of incidence of postoperative hypocalcemia by patient demographics and clinical characteristics.

Variable^a^	Normocalcemic (*n* = 112,962)	Hypocalcemic (*n* = 6,605)	*P* value
Age, mean (95% CI), *y*	50.8 (50.7–50.9)	48.0 (47.6–48.4)	<.001
Gender			<.001
Female	90,409 (80.9)	5,603 (85.1)	
Male	21,380 (19.1)	983 (14.9)	
Ethnicity			<.001
White	61,240 (71.2)	3,568 (70.3)	
Black	10,895 (12.7)	527 (10.4)	
Hispanic	7,282 (8.5)	566 (11.2)	
Asian or Pacific Islander	3,615 (4.2)	240 (4.7)	
Native American	338 (0.4)	16 (0.3)	
Other	2,688 (3.1)	159 (3.1)	
Principal procedure			<.001
Total thyroidectomy	42,413 (37.6)	4,217 (63.9)	
Unilateral thyroid lobectomy	45,189 (40.0)	857 (13.0)	
Thyroidectomy with unilateral neck dissection	2,215 (2.0)	372 (5.6)	
Thyroidectomy with bilateral neck dissection	553 (0.5)	169 (2.6)	
Complete substernal thyroidectomy	2,674 (2.4)	285 (4.3)	
Partial substernal thyroidectomy	1,543 (1.4)	55 (0.8)	
Substernal thyroidectomy, not otherwise specified	101 (0.1)	<10 (0.1)	
Other: isthmectomy, partial thyroidectomy not otherwise specified	18,274 (16.2)	643 (9.7)	
Length of stay, mean (95% CI), *d*	1.85 (1.8–1.9)	3.33 (3.3–3.4)	<.001
Hospital type			.008
Teaching	63,529 (56.3)	3,605 (54.6)	
Nonteaching	49,352 (43.7)	2,997 (45.4)	
Neoplasm of thyroid gland			<.001
Malignant	33,228 (60.0)	3,050 (85.3)	
Benign	22,129 (40.0)	524 (14.7)	

^a^Continuous variables are presented as mean (95% CI); categorical variables are presented as *n* (%).

**Table 3:** Predictors of inpatient hypocalcemia after thyroidectomy.

Risk factor	Odds ratio (95% CI)	*P* value
Age (per year)	0.99 (0.99-0.99)	<.001
Female gender	1.62 (1.45–1.80)	<.001
Ethnicity		
White	1.00	—
Black	0.77 (0.64–0.91)	.003
Hispanic	1.20 (1.06–1.36)	.003
Asian or Pacific Islander	1.21 (1.02–1.44)	.03
Native American	0.79 (0.40–1.56)	.49
Other	0.97 (0.78–1.22)	.80
Principal procedure		
Unilateral thyroid lobectomy	1.00	—
Total thyroidectomy	3.72 (3.27–4.24)	<.001
Thyroidectomy with unilateral neck dissection	5.58 (4.65–6.70)	<.001
Thyroidectomy with bilateral neck dissection	9.42 (7.40–11.99)	<.001
Complete substernal thyroidectomy	3.54 (2.70–4.66)	<.001
Partial substernal thyroidectomy	1.48 (0.75–2.92)	.26
Substernal thyroidectomy, not otherwise specified	6.13 (1.09–34.45)	.04
Other: isthmectomy, partial thyroidectomy not otherwise specified	1.37 (1.13–1.67)	.001
Length of stay (per day)	1.12 (1.11–1.13)	<.001
Nonteaching hospital	1.28 (1.18–1.39)	<.001
Malignant neoplasm of thyroid gland	1.99 (1.76–2.26)	<.001

**Table 4:** Postoperative hypocalcemia after thyroidectomy among multicenter patient cohorts.

Study	Years	Location	Patients (*n*)	Hypocalcemia incidence (%)		Hypocalcemia definition	Independent risk factors
				Overall	TT		
Rosato et al. [16]	1995–2000	Italy	14,934	10.0	14.0	Symptomatic	Thyroid cancer
Hundahl et al. [17]	1996	USA	5,354	10.0	12.4	Study protocol code	NA
Bergenfelz et al. [18]	2004–2006	Sweden	3,660	NA	9.9	Required vitamin D at 1–6 weeks after surgery	Female gender, neck dissection, previous thyroidectomy, resected parathyroid glands, low preoperative serum calcium
Gourin et al. [19]	1990–2009	USA (Maryland)	21,270	NA	10.0	ICD-9	Thyroid cancer, total thyroidectomy, neck dissection, low-volume surgeons
Current study	1998–2008	USA	119,567	5.5	9.0	ICD-9	As described

TT: total thyroidectomy; NA: not available; ICD-9: International Classification of Diseases, Ninth Revision.
